# Behavioural response to heterogeneous severity of COVID-19 explains temporal variation of cases among different age groups

**DOI:** 10.1098/rsta.2021.0119

**Published:** 2022-01-10

**Authors:** Benjamin Steinegger, Lluís Arola-Fernández, Clara Granell, Jesús Gómez-Gardeñes, Alex Arenas

**Affiliations:** ^1^ Departament d’Enginyeria Informàtica i Matemàtiques, Universitat Rovira i Virgili, Tarragona 43007, Spain; ^2^ Department of Condensed Matter Physics, University of Zaragoza, Zaragoza E-50009, Spain; ^3^ GOTHAM Lab—BIFI, University of Zaragoza, Zaragoza E-50018, Spain

**Keywords:** COVID-19, spreading, prophylaxis

## Abstract

Together with seasonal effects inducing outdoor or indoor activities, the gradual easing of prophylaxis caused second and third waves of SARS-CoV-2 to emerge in various countries. Interestingly, data indicate that the proportion of infections belonging to the elderly is particularly small during periods of low prevalence and continuously increases as case numbers increase. This effect leads to additional stress on the health care system during periods of high prevalence. Furthermore, infections peak with a slight delay of about a week among the elderly compared to the younger age groups. Here, we provide a mechanistic explanation for this phenomenology attributable to a heterogeneous prophylaxis induced by the age-specific severity of the disease. We model the dynamical adoption of prophylaxis through a two-strategy game and couple it with an SIR spreading model. Our results also indicate that the mixing of contacts among the age groups strongly determines the delay between their peaks in prevalence and the temporal variation in the distribution of cases.

This article is part of the theme issue ‘Data science approaches to infectious disease surveillance’.

## 1. Introduction

The study of the interplay between human behaviour and the spreading of epidemics has received great attention since the early 2000s [1–3] and is fundamental in many modelling approaches to the spread of SARS-CoV-2 [4]. Particular attention has been paid to how individuals decide to adopt protective measures. Pioneer results from the study of vaccine uptake have shown that the voluntary adoption may cause subsequent outbreaks to occur [5]. Similar findings have been obtained by coupling the epidemic dynamics with the spread of awareness, awareness kernels, or payoff-based considerations regarding the adoption of prophylactic measures [6–10].

Consequently, already from the first epidemic waves caused by the entry of SARS-CoV-2 in different regions of the world, it was clear that changes in behavioural patterns would determine the course of subsequent waves. Actually, early analysis that incorporated the effect of human behaviour and tailored their models for the characteristics of SARS-CoV-2 showed an equivalent phenomenology [11–15]. Finally, the eventuality of a second and subsequent waves fulfilled itself in a series of countries. It was empirically confirmed that, together with seasonality, human behaviour was a substantial factor that caused the emergence of second waves. In particular, individuals continuously relaxed their prophylactic behaviour after the first wave, as can be observed from increased mobility levels [16].

**Figure 1.**
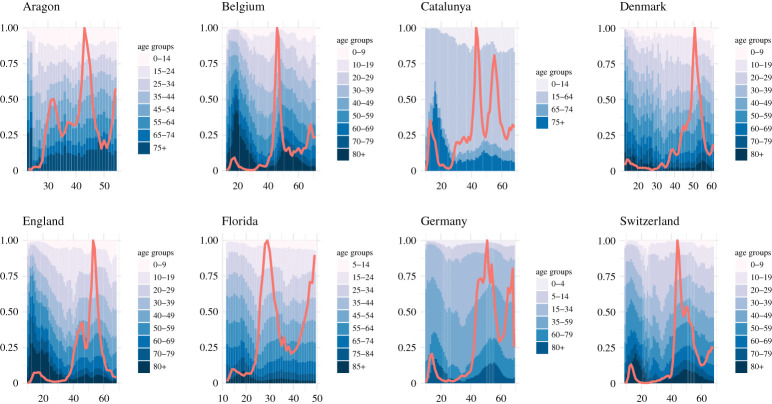
Weekly aggregated distribution of reported cases among the age groups for a variety of regions and countries [17–24]. The x-axis indicates time by numerating the weeks of 2020 and continuing after 2021. The red line shows the evolution of the average weekly incidence, which serves as a proxy for prevalence, normalized to the peak incidence. (Online version in colour.)

**Figure 2.**
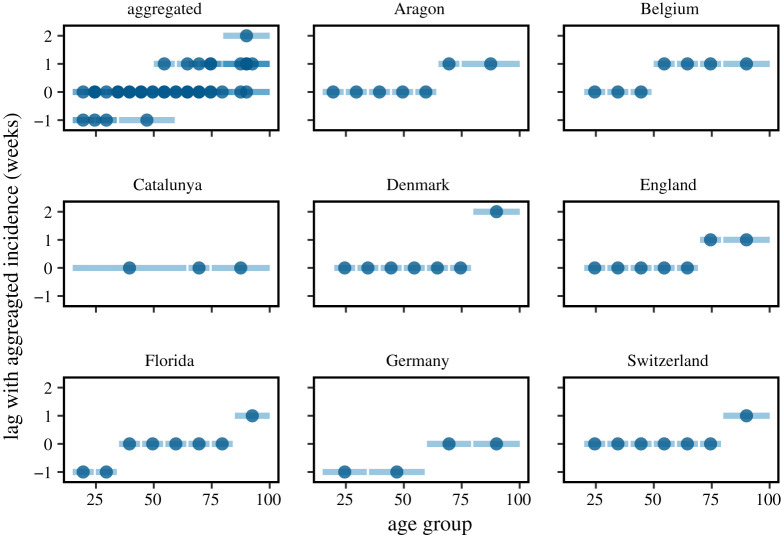
Temporal difference between the peak in the absolute number of infections of a particular age group and the aggregation over all age groups. Lines show the range of the age groups, while dots indicate the mean between the limits. For all countries and regions, we show the second peak. An exception is Germany, whose second peak coincides with the Christmas holidays which made reporting unreliable, wherefore we show the delay for the first wave. (Online version in colour.)

While the emergence of subsequent waves is well established through modelling, we observe additional phenomena that are yet to be captured from a theoretical perspective. Figure 1 shows the evolution of the distribution of reported cases over age groups for various countries and regions. Interestingly, we observe a substantial increase of cases attributed to older age groups with the arrival of a subsequent wave. The fraction of cases attributed to the older age groups then peaks substantially after the peak in the aggregated number of reported cases. An equivalent temporal variation can be observed when considering the fraction of fatalities attributed to nursing homes [17]. Furthermore, figure 2 shows that in various cases also the absolute number of infections in the older age groups peaks with a delay of one week or more with respect to the younger age groups. We observe a clear trend that the prevalence in the elder age groups peaks later. In particular, with the exception of Catalunya, the age group 80+ exhibits a significant delay with respect to the younger age groups across all countries and regions. The same delay was previously observed for fatalities instead of case numbers [25].

In this study, we provide a simple mathematical model which exhibits all the aforementioned phenomenology by considering different age groups, i.e. groups at different risk of a severe course of the disease. We model the individual adoption of prophylactic measures, such as social distancing, increased hygiene or the wearing of face masks, using a game-theoretic approach. Importantly, we explicitly include the heterogeneity regarding the severity of a SARS-CoV-2 infection and how this heterogeneity induces a more cautious behaviour in the risk group. The behavioural model is then coupled to the evolution of a spreading dynamics. This framework allows us to qualitatively understand and mechanistically explain the emergence of the temporal variation over age groups as well as the observed delayed peak in prevalence.

## 2. Model

For the sake of simplicity, we describe the epidemic spreading by a standard SIR model, i.e. considering only susceptible, infected and recovered epidemic compartments. Accordingly, we disregard the possibility of reinfection. In addition, since we do not consider how contact tracing and isolation may prevent the spread of SARS-CoV-2, it is not necessary to differentiate among pre-symptomatic, asymptomatic and symptomatic infectiousness.

Behaviourally, the response to SARS-CoV-2 has been, and continues to be, varied. At the individual level, the adoption of prophylactic measures largely determines the course of the epidemic. Also, central actors, such as public health authorities [26–28], and external factors, for example seasonality [29,30], influence the spread of the epidemic. In addition, all these levels depend on each other. Modelling the interplay among central actors, seasonality and individual actions from a behavioural perspective is very challenging. Nevertheless, here we show that focusing solely on the behavioural response at the individual level proves to be sufficient to explain the relevant phenomenology observed from data.

The response at the individual level mainly consists of the adoption of prophylactic measures such as social distancing, reduction of contacts, adoption of hygiene, attachment to ventilation or the use of face masks. From a mathematical perspective, in a continuous time mean field model, all these measures simply reduce the transmission rate either through a reduction of contacts or the transmission probability and are thus essentially equivalent [31]. This equivalence enables us to subsume the different measures into one category: the adoption of prophylactic measures. In our model, we assume that the adoption of prophylactic measures is driven by the risk of infection and the potentially severe consequences of such an infection, personally or in one's private sphere.

In this sense, we describe the decision to adopt prophylactic measures as a two-strategy game, reducing, for simplicity, the behavioural response to a binary choice: individuals either adopt prophylactic measures (P) or not (*NP*). The associated payoffs of each strategy express the trade-off between the infection cost, T, modulated by the infection risk and the adoption cost of prophylactic measures, c. Due to the existence of individuals at higher risk of a severe course of COVID-19, we divide the population into G different groups with different infection costs $T_{i}$. For the sake of generality, we will also consider all other behavioural parameters specific to each group. Accordingly, we define the payoffs of group i as follows:

2.1 $$P_{i}^{\text{NP}}=-T_{i}\sum_{j=1}^{G}f_{j}(I_{j}^{\text{NP}}+I_{j}^{P})$$

and

2.2 $$P_{i}^{P}=-c_{i}-T_{i}(1-\gamma_{i})\sum_{j=1}^{G}f_{j}(I_{j}^{\text{NP}}+I_{j}^{P}).$$

The sum expresses the global infection risk, i.e. the aggregated fraction of infected individuals, with $f_{i}$ representing the fraction of the population belonging to group i. The variables $I_{i}^{P}$ and $I_{i}^{\text{NP}}$ refer to the fraction of infectious individuals in group i that adopt and do not adopt prophylactic measures, respectively. For protected individuals, we modulate the infection risk by the efficacy of the prophylactic measures, $\gamma_{i}$. Given the payoffs, individuals belonging to group i adopt the more beneficial strategy according to the transition probabilities

2.3 $$\Gamma_{i}^{P\to \text{NP}}=\Theta (P_{i}^{\text{NP}}-P_{i}^{P})\frac{P_{i}^{\text{NP}}-P_{i}^{P}}{c_{i}+T_{i}}$$

and

2.4 $$\Gamma_{i}^{\text{NP}\to P}=\Theta (P_{i}^{P}-P_{i}^{\text{NP}})\frac{P_{i}^{P}-P_{i}^{\text{NP}}}{c_{i}+T_{i}}.$$

The letter Θ represents the Heaviside function and guarantees that only the more successful strategy is adopted. The denominator, which corresponds to the maximal difference in payoff, serves to bound the probabilities. When the dynamics evolve, individuals update their strategy according to the adoption—selection—rate $\alpha_{i}$ with the probabilities $\Gamma_{i}^{P\to \text{NP}}$, $\Gamma_{i}^{\text{NP}\to P}$. Finally, by coupling the behavioural dynamics to the SIR spreading model, the system of differential equations can be written as follows:

2.5 $$\begin{array}{r} {\dot{S}}_{i}^{\text{NP}}=-\beta_{i}S_{i}^{\text{NP}}\sum_{j=1}^{G}C_{ij}(I_{j}^{\text{NP}}+(1-\gamma_{j})I_{j}^{P})+\alpha_{i}S_{i}^{P}\Gamma_{i}^{P\to \text{NP}}-\alpha_{i}S^{\text{NP}}\Gamma_{i}^{\text{NP}\to P} \end{array}$$

2.6 $$\begin{array}{r} {\dot{S}}_{i}^{P}=-\beta_{i}S_{i}^{P}(1-\gamma_{i})\sum_{j=1}^{G}C_{ij}(I_{j}^{\text{NP}}+(1-\gamma_{j})I_{j}^{P})+\alpha_{i}S_{i}^{\text{NP}}\Gamma_{i}^{\text{NP}\to P}-\alpha_{i}S^{P}\Gamma_{i}^{P\to \text{NP}} \end{array}$$

2.7 $$\begin{array}{r} {\dot{I}}_{i}^{\text{NP}}=\beta_{i}S_{i}^{\text{NP}}\sum_{j=1}^{G}C_{ij}(I_{j}^{\text{NP}}+(1-\gamma_{j})I_{j}^{P})-\mu_{i}I_{i}^{\text{NP}} \end{array}$$

2.8 $$\begin{array}{r} {\dot{I}}_{i}^{P}=\beta_{i}S_{i}^{P}(1-\gamma_{i})\sum_{j=1}^{G}C_{ij}(I_{j}^{\text{NP}}+\Gamma_{i}I_{j}^{P})-\mu_{i}I_{i}^{P} \end{array}$$

2.9 $$\begin{array}{r} \;\text{and}\;{\dot{R}}_{i}=\mu_{i}(I_{i}^{\text{NP}}+I_{i}^{P}). \end{array}$$

In the aforementioned equations, the parameters $\beta_{i}$ and $\mu_{i}$ refer to the infection and recovery rate, respectively. The matrix C represents the contact matrix and controls the mixing among the different groups. The variables $S_{i}^{P}$ and $S_{i}^{\text{NP}}$ refer to the fraction of susceptible individuals of group i that adopt and do not adopt prophylactic measures, respectively. The variable $R_{i}$ subsumes the fraction of recovered individuals in group i.

## 3. General phenomenology for a homogeneous population

**Figure 3.**
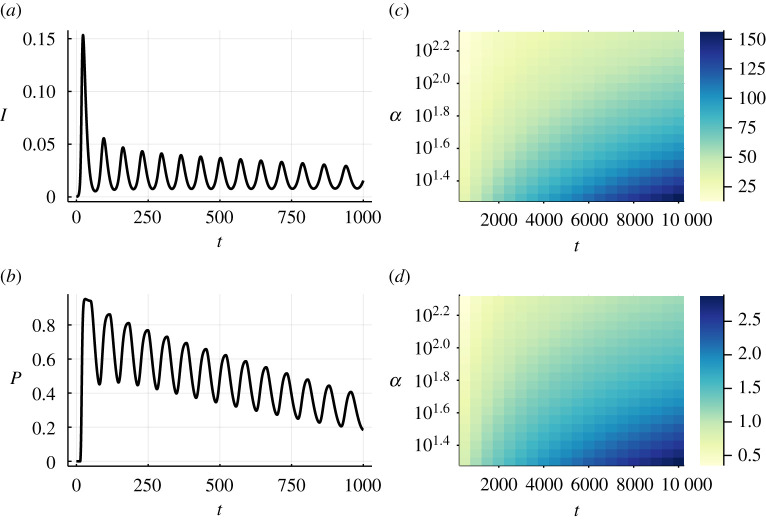
(*a*) Prevalence or fraction of infected individuals, $I=I^{\text{NP}}+I^{P}$ and (*b*) fraction of protected individuals $P=S^{P}+I^{P}$ evolving in time for a single realization of the process, with initial conditions $I^{\text{NP}}(0)=10^{-5}$, and the remaining fraction is attributed to the compartment $S^{\text{NP}}(0)$, with fixed α=50 and T=2000. (*c*) Average time span and (*d*) amplitude of the epidemic waves depending on the infection cost, T, and adoption rate, α. All results are obtained by integrating equations (2.5)–(2.9) with fixed Δt=0.005 and parameters β=0.6, μ=0.2, γ=0.4 and c=1. (Online version in colour.)

Before focusing on the effect of different risk groups, we analyze the general phenomenology for a homogeneous population. In this scenario, we consider only one group of well-mixed individuals with constant infection cost T and the same epidemiological parameters. We study the dynamics of the system by numerically integrating the differential equations [32]. In figure 3*a*, we present an exemplary trajectory for the prevalence, I. We note how subsequent waves arise as occurs for other models that couple disease dynamics with the behavioural response [1,3,5–7,9]. These subsequent waves emerge from a constant strengthening and weakening of the prophylactic measures (figure 3*b*), which cause the reproduction number to oscillate around one. Actually, a series of previous studies have shown as to how the reproduction number oscillates around one, whenever the prophylactic behaviour depends on the global infection risk [5,6,9], which is also the case here.

The emergence of the observed oscillatory behaviour is conditioned by the activation of a behavioural response in the population. While it is well understood that the epidemic threshold is not affected by the behavioural response [9], the protection threshold at which prophylaxis starts to be adopted strongly depends on the reach of the epidemic outbreak. In particular, there only exists a behavioural response if $P^{P}>P^{\text{NP}}$, i.e. if the benefits of adopting prophylactic measures are greater than not adopting them. To satisfy this condition, a critical prevalence, $I^{*}$, is required. From equations (2.1) and (2.2), we obtain that

3.1 $$I^{*}=\frac{c}{\gamma T}.$$

In other words, the maximum prevalence, $I^{\text{max}}$, during an outbreak must exceed $I^{*}$ to observe prophylactic behaviour. Since we consider a homogeneous population, the maximal prevalence is given by the one of the standard SIR models [31]

3.2 $$I^{\text{max}}=1-\frac{1}{R_{0}}(1+\ln(R_{0})).$$

The variable $R_{0}=\beta /\mu$ refers to the basic reproduction number of the disease. Combining equations (3.1) and (3.2), we find the critical ratio between the protection and infection cost such that a behavioural response emerges:

3.3 $$\frac{c}{T}<\gamma \left[1-\frac{1}{R_{0}}(1+\ln(R_{0}))\right].$$

If one had several groups with different infection/protection costs but with the same transmission rates, $\beta_{i}$, and random mixing, the same condition would hold. In this case, the group with the lowest ratio $c_{i}/T_{i}$ would initiate the behavioural response.

Further inspection of figure 3*a*,*b* shows that, while the average prevalence stays relatively constant over time, the protection level slowly decreases. The average prevalence is almost constant since the system oscillates around the behavioural equilibrium $P^{\text{NP}}=P^{P}$. For $P^{P}>P^{\text{NP}}$, prophylaxis increases and vice-versa. However, due to the increasing immunity—intrinsic to the SIR model—less prophylaxis is needed to maintain the prevalence level, wherefore the average protection level decreases over time.

In addition, we note that the first wave leads to the highest prevalence. However, this is not always the case and strongly depends on the initial condition. When the initial fraction of protected individuals is low, the amplitude of subsequent waves continuously decreases as shown in figure 3*a*. However, if initially prophylaxis is widespread, the second wave leads to a higher peak in prevalence than the first one and the amplitude subsequently decreases. Furthermore, for an even higher initial protection level, the amplitude can rise for subsequent waves before the decrease starts. This dependence on the initial condition might explain cases such as Germany or Switzerland, where the second wave of SARS-CoV-2 reached higher infection levels than the first one. In analogy to the initial condition of the model, this may be explained by relatively high protection levels at the start of the first wave due to the experience from Italy and Spain. However, in this study, we focus on the regime where the first wave is the dominant one and leave a detailed study on the initial conditions for future work.

To conclude the study of the homogeneous population, we analyze the effect of the parameters related to behavioural aspects of the model. In particular, we study the influence of the adoption rate, α, and the infection cost, T, on the epidemic dynamics. For a detailed analysis of the remaining parameters, we refer the reader to reference [9]. Figure 3*c*,*d* show that both a larger infection cost and a lower adoption rate increase the time span between subsequent waves (the period) as well as their amplitude. Intuitively, the slower or more delayed the reaction is, the more time it will take to get subsequent waves under control, wherefore their amplitude also increases. Overall, this is the basic phenomenology in the absence of any risk groups, which we will explore in the next section.

## 4. Distinct risk groups

Although we have so far formulated the model in general terms, i.e. by considering all the parameters inherent to every group G, we will focus on the case of two groups in the following. The reduction in the number of groups simplifies the model while preserving the key phenomenology at hand, namely the existence of a diverse risk group for which the course of COVID-19 is highly probable to be severe [33,34]. In addition, the presence of risk factors correlates widely with age, resulting in a strong and monotonous age dependence of the infection fatality ratio (IFR), which justifies our modelling in terms of young and elderly individuals [35–37].

The higher mortality for the elderly is also reflected in their behaviour. Even though the perceived and actual risk do not always match with human behaviour, a series of studies have shown that the elderly were more cautious, i.e. showed intensified social distancing through a stronger reduction in mobility [38–40], contacts [41,42] or credit card expenses [43] than the rest of the population. This is also reflected in a higher seroprevalence in the younger age groups [36].

The higher IFR for elderly combined with the observed stronger adherence to prophylactive measures supports the introduction of different infection costs, $T_{i}$. Ideally, one could work with a distribution of infection costs or one for each age strata. However, such an approach would come at the cost of simplicity and could mask the key ingredients that contribute to the basic phenomenology. For this reason, we will categorize the population into low- and high-risk groups, referred to as young (Y) and old (O), that have associated infection costs $T_{Y}$ and $T_{O}$, respectively. Later in the article, we will comment on how the division into more subgroups could affect the dynamics. Similar to the infection cost, one could imagine distinguishing the adoption cost, $c_{i}$, the transmission rate, $\beta_{i}$, or the efficacy of the prophylactic measures $\gamma_{i}$ between the two groups. However, for the sake of simplicity, we will consider the parameters $c_{i}$, $\beta_{i}$ and $\gamma_{i}$ equal in the two groups, as well as the recovery probability $\mu_{i}$ and the adoption rate $\alpha_{i}$. We parametrize the interaction between the two groups, i.e. the 2×2 contact matrix C, with a mixing rate ν. To be more precise, given the fraction of old, $f_{O}$ and young individuals, $f_{Y}$, in the population, the four elements of the contact matrix read as follows:

4.1 $$\begin{array}{r} C_{YO}=\nu f_{O} \end{array}$$

4.2 $$\begin{array}{r} C_{OY}=\nu f_{Y} \end{array}$$

4.3 $$\begin{array}{r} C_{YY}=1-\nu f_{O} \end{array}$$

4.4 $$\begin{array}{r} \;\text{and}\;C_{OO}=1-\nu f_{Y}. \end{array}$$

For ν=0, the two groups do not interact with each other, while ν=1 corresponds to random mixing. In the following, we will assume that 20% of the population belong to the group at risk, i.e. $f_{O}=0.2$ and $f_{Y}=0.8$.

**Figure 4.**
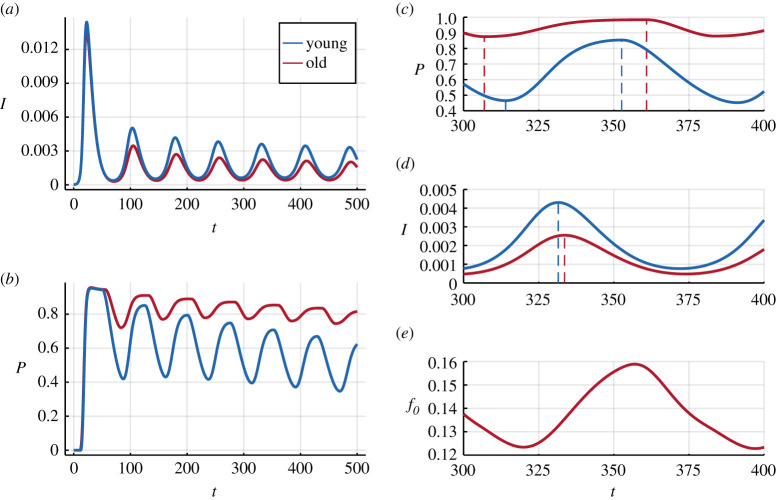
(*a*) Prevalence or fraction of infected individuals, $I_{i}=I_{i}^{\text{NP}}+I_{i}^{P}$ and (*b*) fraction of protected individuals $P_{i}=S_{i}^{P}+I_{i}^{P}$ in the old (red) and young (blue) groups, evolving in time, with initial conditions fixed as in figure 3 for both groups. The adoption rate is fixed at α=50 and the infection costs are $T_{O}=2000$ and $T_{Y}=500,$ respectively. (*c*) Fraction of protected individuals and (*d*) fraction of infected individuals in both groups displayed in a shorter time window. (*e*) The fraction of cases belonging to the old group in time. All results are obtained by numerically integrating equations (2.5)–(2.9) using a standard ODE solver, with fixed Δt=0.005, and in both groups, β=0.6, μ=0.2, γ=0.4 and c=1. (Online version in colour.)

In figure 4*a*,*b*, we see that, similarly to a homogeneous population, both groups exhibit recurrent epidemic waves driven by a varying prophylaxis level. Due to the higher infection cost, the old group shows more intense prophylaxis and thus lower prevalence. In terms of the temporal evolution, we observe that the elderly population also starts to increase prophylaxis earlier (dashed lines) as prevalence increases and continues to do so for longer as prevalence decreases. This is shown in figure 4*c*. Furthermore, the variation in the prophylaxis level of the young is more pronounced and thus mainly drives the evolution of the prevalence. As a consequence, in figure 4*d*, we observe how the prevalence of the young group increases more rapidly and spills over to the old group. As a matter of fact, due to the increased prevalence tolerance caused by a lower infection cost of the young group, the reproduction number among the old is always below one. Accordingly, prevalence in the old group solely increases due to the infections from the young group. This mechanism leads to a slight delay in the peak of the old group compared to the young group (dashed lines) as one can see in figure 4*d*. The model exhibits the same delay among the peaks as observed from empirical data, which we presented in figures 1 and 2.

Furthermore, due to the different growth rates, in figure 4*e*, we see a temporal variation in the case distribution, i.e. in the fraction of cases belonging to the old group. This phenomenology was also observed in the data displayed in figure 1. If we look across figure 4*c*–*e*, we see that the fraction of cases belonging to the old group (figure 4*e*) is minimal when prevalence is low (figure 4*d*), at the point where the prophylaxis level differs substantially between the two groups (figure 4*c*). However, as prevalence increases (figure 4*d*), the prophylaxis level between the two groups becomes closer (figure 4*c*), wherefore also the growth rates of the epidemic in both groups differ less (figure 4*d*) and the fraction of cases belonging to the old group approaches the population distribution (figure 4*e*). This dynamics leads to the temporal variation of the case distribution.

Overall, results in figure 4 show how the varying infection risk among the two groups, which translates into a heterogeneous behavioural reaction, can explain the phenomenology—the time varying case distribution as well as the delay among prevalence peaks—that we observed in the data. Furthermore, we checked that imposing different contact rates, $\beta_{i}$, when the infection cost are the same in each group, does not lead to any of the two phenomena. On the other hand, the phenomenology holds also when contact rates differ together with the infection cost. In this sense, at least under the framework of this model, the behavioural heterogeneity is truly the key factor that reproduces qualitatively the real-world observations.

As pointed out earlier, we simplified the model and separated the population into low- and high-risk groups. However, one could also introduce additional divisions or directly make use of the nine age groups as in figure 1. If one assumes that the infection cost progressively increases with age the general phenomenology is very similar. Groups will reach their epidemic peak subsequently according to their infection cost. Similarly, the fraction of infections in the groups with high infection cost increases towards the peak and is particularly small during times of low prevalence. Since the phenomenology is robust regarding the division into additional groups but hinders clear and concise visualization, we refrained from presenting it.

To which extent the phenomenology described earlier is pronounced depends on the parameters. In the following, we analyze the effect of the infection cost and the mixing rate between the groups and also the dependence on the adoption rate, unveiling a few interesting effects.

**Figure 5.**
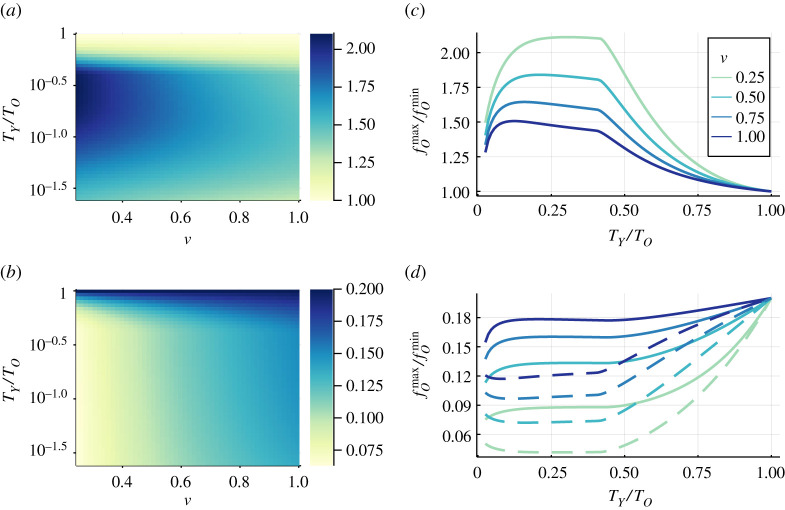
Top: Ratio between maximum, $f_{O}^{\max}$, and minimum, $f_{O}^{\max}$, in the variation of the case distribution, i.e. in the fraction of infected individuals belonging to the old group, $f_{O}$, in time depending on the mixing rate between the groups, ν, and the ratio of infection costs $T_{Y}/T_{O}$ (*a*). We also show the dependence on the infection cost in the young group, when the infection cost for the old group is fixed, $T_{O}=2000$, for four choices of the mixing rate (*c*). Bottom: Average between maximum, $f_{O}^{\max}$, and minimum, $f_{O}^{\min}$, in the variation of the case distribution, $f_{O}$ depending on the mixing rate between the groups, ν, and the ratio of infection costs $T_{Y}/T_{O}$ (*b*). We also show how the maximal (solid) and minimal (dashed) fraction of cases belonging to the old group depends again on the infection cost of the young group $T_{Y}$, when the infection cost for the old group is fixed, $T_{O}=2000$, for the same choices of the mixing rate (*d*). All results are obtained by numerically integrating equations (2.5)–(2.9) using a standard ODE solver, with fixed Δt=0.005, and, in both groups, α=0.50, β=0.6, μ=0.2, γ=0.4 and c=1, and initial conditions $I^{\text{NP}}(0)=0.0015$ in both groups. (Online version in colour.)

In figure 5*a*,*c*, we observe that the variation in the case distribution non-trivially depends on the ratio between the infection costs of the two groups for the whole range of the mixing rate ν. If the infection cost of the young group is too low, the protection level does not significantly vary. Accordingly, the case distribution stays pretty stable. On the other hand, if the infection cost of the young approaches that of the old, we return to the homogeneous case, where the case distribution does not vary either. The competition between those two processes causes the maximum in the relative variations of the case distribution in time.

Interestingly, the lower the mixing rate, the more pronounced is the case variation over time. In contrast, as we show in figure 5*b*,*d*, a lower mixing rate decreases prevalence among the old due to the better structural shielding. This is particularly relevant since a good shielding reduces prevalence among the risk group but leads to substantial variation of the case distribution. These variations then may cause an increase in IFR as well as in the infection hospitalization rate towards the peak of infection, which would put additional stress on the health care system. Finally, again here, we observe how the case distribution approaches the population structure as the infection cost of the old approaches that of the young.

**Figure 6.**
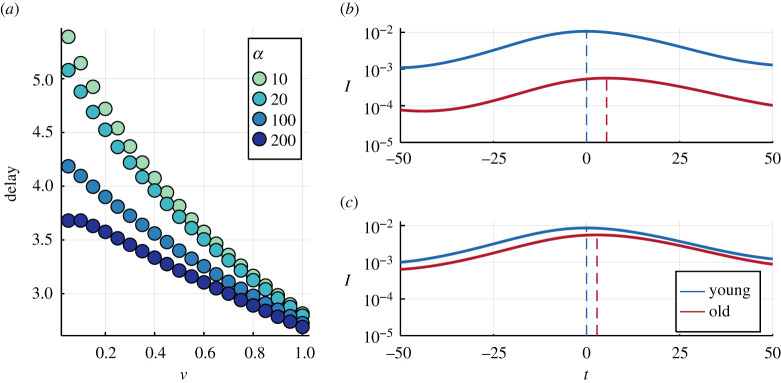
(*a*) Dependence on the mixing rate for four choices of the adoption rate α. All results are obtained by numerically integrating equations (2.5)–(2.9) using a standard ODE solver, with same initial conditions as in figure 5, with fixed Δt=0.005, and infection costs $T_{O}=2000$ and $T_{Y}=500$. In both groups, α=10, β=0.6, μ=0.2, γ=0.4 and c=1. Right: Zoom into the temporal evolution of the fraction of infected individuals for (*b*) ν=0.05 and (*c*) random mixing, ν=1.0. Dashed lines indicate the position of the maximum. The adoption rate was fixed as α=10. Time, t, was shifted such that the peak of the age group young is located at zero. (Online version in colour.)

Finally, in figure 6*a*, we see how the delay between the peaks monotonously decreases with mixing rate as well as with the adoption rate. In particular, a more modular contact matrix (lower ν) delays the increasing prevalence in the young group from propagating into the risk group. Figure 6*b*,*c* show the temporal evolution, for two values of the mixing rate between the young and old population, ν. For low mixing (figure 6*b*), we observe a larger delay in the epidemic peak than in the case of a high mixing (figure 6*c*). This means that a better overall shielding of the risk group (lower mixing) (figure 6*b*) may lead to an unforeseen, late peak in prevalence among the risk group after the arrival of a wave. As previously shown in figure 3*c*, a higher adoption rate, α, decreases the intervals at which waves pass and thus leaves less time for the prevalence to propagate into the risk groups. Consequently, the delay between the peaks shrinks as individuals adopt their behaviour faster.

## 5. Conclusion

By analyzing the available information of confirmed SARS-CoV-2 infections, we have shown how the case distribution among age groups temporally varies through time. Furthermore, we highlighted how the cases among the older age groups—in relative as well as absolute terms—peak later than the total number of cases. This phenomenology is observed for subsequent waves as well as across a diverse set of countries and regions.

We proposed a minimal, mechanistic model that exhibits the same phenomenology. The disease is controlled through the voluntary adoption of prophylactic measures, which we modelled as a two-strategy game. Within this framework, the key factor is the heterogeneous risk of a severe course of a disease that widely correlates with age. The varying severity of the disease translates into a heterogeneous behavioural reaction. To be more precise, the group at lower risk drives the infections as their adoption of prophylaxis is not as widespread and occurs later. The changing difference in prophylaxis between the high- and low-risk group leads to a temporal variation of the case distribution. Furthermore, as the low-risk group drives the infections towards their peak, maximal prevalence is reached with a delay among the risk group. Both phenomena match the observations from the data.

There is an interest in this study from a theoretical point of view, since a minimal model is able to exhibit the same phenomenology as the reported data. More importantly, the study highlights various points relevant for the public health policies of the pandemic. Our results indicate that an initial low fraction of infections among the risk group can lead to an underestimation of the potential future stress on the health care system. In fact, we observed such an underestimation during the summer months of 2020. In this period, the reported cases were mainly among younger people, leading to a reduced stress on health care systems. Consequently, the public discourse advocated tolerating higher prevalence levels, i.e. restoring social fabric while coexisting with virus transmission. However, along with seasonal effects, the resulting behavioural relaxation led to an increase in prevalence among the at-risk group, resulting in the emergence of severe second waves in several countries around the world.

There are several limitations to this study. We did not include the impact of seasonal effects and government policies on the behavioural response to the epidemic. Such an approach would require more detailed, large-scale behavioural data and a deeper understanding of human behaviour as well as the specifities of the virus. Nevertheless, already minimal data, such as age-stratified case numbers, allowed us to observe regularities in the infections patterns. In addition to the data analysis, modelling efforts are necessary to unveil the mechanisms behind the observations. In this sense, more publicly available data regarding the behavioural response would allow us to include the behavioural response dynamically into epidemic models. Such a refined approach from a behavioural point of view is crucial to advance from conditional scenarios to actual predictions.

## Data accessibility

All data used in this study is publicly available and can be accessed through Refs. [15–22].
